# Solidarity and Voluntarism Amid the COVID-19 Pandemic: Skin Cancer Screening for Blood Donors

**DOI:** 10.5826/dpc.1103a80

**Published:** 2021-05-20

**Authors:** Katerina Grafanaki, Sophia Georgiou, Alexander J. Stratigos

**Affiliations:** 1 Department of Dermatology, University Hospital of Patras, School of Medicine, University of Patras, Greece; 2 Department of Dermatology-Venereology, Andreas Syggros Hospital, National and Kapodestrian University of Athens, Greece

**Keywords:** skin cancer screening, volunteer blood donors, teledermatology, Red Cross, public health, solidarity

Melanoma is the deadliest skin cancer, and its incidence is increasing globally. It is preventable, and indeed increased sun awareness campaigns and early diagnoses through skin cancer screening programs for the general population have contributed to decreasing mortality from melanoma. Many of the volunteer activities involved in raising public awareness about melanoma are provided by dermatological societies and volunteer dermatologists who are often not sufficiently credited for their contributions. The recipients of these free services include blood donors and emergency service providers (eg, Hellenic Red Cross Samaritans).

Although efforts over the past decade have led to a significant reduction in skin cancer, activities have been suspended and, in many cases, disrupted since the outbreak of coronavirus disease 2019 (COVID-19) created a public health emergency of international concern. National health systems are overburdened, and patients are avoiding specialist visits due to fears of becoming infected with the SARS-CoV-2 virus while attending a medical clinic. As a result, a 68.61% reduction in skin cancer diagnoses has been reported in the UK [1]. During the strict government measures to contain the spread of SARS-CoV-2 and minimized the number of COVID-19 cases, dermatologists have limited their activities to dermatological emergencies and have opted to practice teledermatology for less urgent issues.

Concurrently, another major disruption due to the COVID-19 pandemic is a shortage of blood for transfusions. Every 2 seconds, a patient in the United States needs a red blood cell transfusion; almost 5,000 platelet units and 6,500 units of plasma are required daily. Plasma containing antibodies against SARS-CoV-2 could be transfused into severely ill patients with COVID-19 [2]. Importantly, lack of ethnic diversity within the donor pool and rare blood types should be acknowledged [3]. During the pandemic, 86,000 American Red Cross (ARC) blood drives were cancelled, whereas in Europe and Asia blood banks continue to play a vital role in ensuring an uninterrupted supply of blood and blood components [3]. An opportunity to donate is an opportunity to save a life. The ARC is offering free SARS-CoV-2 antibody testing to blood donors [2]. The International Federation of the Red Cross is an impartial, neutral, and independent organization whose mission is to assist in the response to humanitarian emergencies such as epidemics [3].

Since 1985, the American Academy of Dermatology has been offering free skin cancer screening. Volunteer dermatologists have performed more than 2.8 million screenings and detected more than 31,500 suspected melanomas and 278,000 suspicious lesions, saving countless lives [4]. Skin Cancer Awareness Month (United States), Euromelanoma (Europe), and similar campaigns on other continents, which have a significant impact on early diagnosis of melanoma, were either mostly realized via internet and were mass media-based or were cancelled, depending on the country’s restrictions. While the pandemic may represent a threat to many aspects of our existence, dermatologists can show a humanitarian face through voluntary skin cancer screening of life-saving volunteer blood donors.

Early detection of melanoma is facilitated by teledermatology using smartphones [5] and teledermoscopy [6]. These tools increase dermatological access in rural regions and contribute to gender, racial and ethnic equity [7], especially in heterogeneous socioeconomic groups such as blood donors who would otherwise not seek a dermatologist during these times. Virtual melanoma checks, with 94% diagnostic accuracy [8], could eliminate the risk of contracting COVID-19. Patients with potentially malignant lesions can be diagnosed and biopsied. The limitation due to the number of high-risk blood donors to be screened could be overcome through careful selection by a pre-screening questionnaire considering their age, phototype, and familial, personal and sun exposure histories. For example, people who volunteer as lifeguards, a profession with high sun exposure, could be prioritized. Teledermatology could be delivered using either store-and-forward or real-time video technology, or a hybrid of both [9].

“In the presence of epidemics or other danger, I will not allow fear of personal harm to turn me from my duty,” says the Hippocratic Oath. Most of us have not worked through pandemics before, but it is time to embrace humanity and solidarity. Volunteering motivation is important in forming a person’s decision to contribute their time and effort without expecting financial rewards. In a time of deep economic crisis, unemployment and social exclusion, solidarity and non-profit assistance to fellow human beings is an urgent need of both the state and society. Amid the ongoing pandemic, blood donations and skin cancer screening must not be disrupted; they can be continued as long as attention is foremost paid to patient safety.

Supporting volunteer blood donors through voluntary skin cancer screening could be an excellent initiative, bringing people together, giving us the opportunity to abandon the illusion of power and admit our weakness to the ongoing pandemic. Only with humility, solidarity, and insight can we accomplish sustainability despite the challenges. Skin cancer screening of volunteer blood donors could be a high-impact skin cancer prevention initiative, rewarding and motivating for volunteers. The volunteer dermatologist may feel fulfillment from wiping out preventable skin cancer. The blood donor will be motivated and encouraged to continue with their life-saving donations.

In light of a change in dermatology practice, investing in prevention strategies in public health and social protection is the mark of a responsible action policy. The COVID-19 pandemic is probably here to stay and is delaying preventive skin cancer diagnosis and treatment that our health system will struggle to cover. Perhaps this is the impetus to mobilize and do something useful and lifesaving during this public health emergency.
